# Potentially inappropriate prescriptions of antibiotics in geriatric psychiatry—a retrospective cohort study

**DOI:** 10.3389/fpsyt.2023.1272695

**Published:** 2024-01-09

**Authors:** Tabea Pfister, Sebastian Schröder, Johannes Heck, Stefan Bleich, Tillmann H. C. Krüger, Felix Wedegärtner, Adrian Groh, Martin Schulze Westhoff

**Affiliations:** ^1^Department of Psychiatry, Social Psychiatry and Psychotherapy, Hannover Medical School, Hannover, Germany; ^2^Institute for Clinical Pharmacology, Hannover Medical School, Hannover, Germany

**Keywords:** geriatric psychiatry, drug safety, potentially inappropriate medications, antibiotics, antibiotic stewardship

## Abstract

**Introduction:**

Older patients are frequently affected by infectious diseases and adverse drug reactions (ADRs) of consecutively prescribed antibiotics. Particularly within geriatric psychiatry, high rates of potentially inappropriate prescriptions (PIPs) have been described, significantly complicating pharmacological treatment. Therefore, this study aimed to investigate the frequency and characteristics of antibiotic PIPs in geriatric psychiatry.

**Methods:**

Medication charts of 139 patient cases (mean age 78.8 years; 69.8% female) receiving antibiotic treatment on a geriatric psychiatric ward were analyzed. Utilizing previously published definitions of antibiotic PIPs, adequacy of the antibiotic prescriptions was subsequently assessed.

**Results:**

16.3% of all screened patient cases (139/851) received an antibiotic treatment during their inpatient stay. 59.5% of antibiotic prescriptions were due to urinary tract infections, followed by pulmonary (13.3%) and skin and soft tissue infections (11.3%). 46.7% of all antibiotic prescriptions fulfilled at least one PIP criterium, with the prescription of an antibiotic course for more than seven days as the most common PIP (15.3%).

**Discussion:**

Antibiotic PIPs can be considered as a frequent phenomenon in geriatric psychiatry. Especially the use of fluoroquinolones and cephalosporins should be discussed critically due to their extensive side effect profiles. Due to the special characteristics of geriatric psychiatric patients, international guidelines on the use of antibiotics should consider frailty and psychotropic polypharmacy of this patient population more closely.

## 1 Introduction

Antibiotic resistance is a global health-related problem significantly complicating the treatment of infectious diseases and currently causing more than one million deaths per year worldwide (1). Potentially inappropriate prescriptions (PIPs) are a major risk factor for the development of antibiotic resistance (2). PIPs include, for example, the use of unindicated or ineffective antibiotics or the prescription of incorrect doses or a prolonged antibiotic treatment duration (2). In view of this problem, recent years have seen the launch of numerous initiatives to reduce the use of antibiotics. In hospitals, for example, Antibiotic Stewardship (ABS) projects have emerged with the aim of promoting the rational use of antibiotics (3). Meanwhile, their effectiveness has been demonstrated in meta-analyses (4).

Recent evidence suggests that the majority of antibiotic PIPs occur in the inpatient setting (5). Older patients in particular are frequently affected by bacterial infections and, consequently, are frequently exposed to antibiotic PIPs, thus representing an at-risk group (6). In addition, geriatric patients are at particular risk for the occurrence of adverse drug reactions (ADRs) due to physiologically altered pharmacokinetic and pharmacodynamic properties (e.g., lower volume of distribution, increased sensitivity to psychotropic side effects) as well as age-related multimorbidity and associated polypharmacy (7). Therefore, various lists of potentially inappropriate medications for older persons (PIM) have been developed and consented by expert panels. The forerunners were the Beers criteria, developed in the USA in 1991, and the PRISCUS list as its German equivalent, which was revised in 2023 (8, 9). However, during the compilation of PIM lists, the assessment of antibiotic use in older patients has often been neglected. Thus, antibiotic use has been assessed in the past based on guidelines or expert opinions (10). In 2022, Baclet and colleagues were the first to publish a definition of 65 explicit PIP criteria related to antibiotic use in older hospitalized patients (11).

Geriatric psychiatric patients often display various comorbidities, which in turn require interdisciplinary management. Due to sarcopenia and age-associated alterations in immune organ and general physiological functions it was shown that infectious diseases are common in this patient population and cause prolonged hospital stays (12). A rational use of antibiotics in geriatric psychiatry is therefore of paramount importance; however, no data on corresponding prescription characteristics are available in the literature to date.

Therefore, the aim of the present study was to assess the prevalence and characteristics of antibiotic PIPs in geriatric psychiatry. Medication charts, discharge letters, results of blood examinations and antibiograms of patients of a large geriatric psychiatric ward in a German university hospital served as basis for the investigation. The identification and categorization of PIPs was based on the Baclet et al. criteria (11).

## 2 Materials and methods

### 2.1 Ethics approval

This study was approved by the Ethics Committee of Hannover Medical School (No. 10593_BO_K_2022) and adheres to the Declaration of Helsinki (1964) and its later amendments (current version from 2013).

### 2.2 Eligibility criteria

Patients were retrospectively enrolled in the study (i) if they were ≥ 65 years of age, (ii) if they were treated on the geriatric psychiatric ward of the Department of Psychiatry, Social Psychiatry and Psychotherapy of Hannover Medical School between January 2014 and March 2022, (iii) if they received a documented treatment with oral or intravenous antibiotics, and (iv) if they or their legal representative had provided written informed consent that patient-related data could be used for clinical research. Hannover Medical School is a large university hospital and tertiary care referral center in northern Germany. The geriatric psychiatric ward is a 27-bed facility specialized in the treatment and care of older psychiatric patients. The ward focuses on the treatment of all types of psychiatric disorders in older people. However, a particular focus is on the diagnosis and treatment of dementia, although patients with depression or psychotic disorders are also regularly treated. The ward also provides elective as well as emergency treatment of psychiatric diseases.

### 2.3 Medication chart reviews, PIP classification system, and demographic characteristics

Medication charts of enrolled patients were analyzed by an expert panel including specialists in antibiotic treatment (ABS certified physicians and clinical pharmacologists), clinical psychiatry and geriatric medicine. Prescriptions of antibiotics were critically discussed, utilizing patient-related data from discharge letters, as well as results from blood examinations (with special focus on infection parameters like leukocyte counts or C-reactive protein (CRP)) and microbiological routine diagnostics. The use of all prescribed oral and intravenous antibiotics (topical antibiotics were excluded) was assessed with the aid of the explicit PIP definitions for antibiotics by Baclet et al. (11).

The objective of the categorization by Baclet et al. (11) was to establish expert-based explicit definitions of antibiotic PIPs in hospitalized geriatric patients. To this end, Baclet et al. (11) conducted a qualitative, multicenter, focus-based study, leading to 65 definitions of antibiotic PIPs classified into 18 domains. Most PIPs affect the prescription of fluoroquinolones, amoxicillin-clavulanic acid, and cephalosporins. Furthermore, definitions of inappropriate prescriptions mostly referred to antibiotic misuse in specific organ systems. In this regard, antibiotic PIPs frequently concerned the use in urinary tract infections (UTIs) like the prescription of nitrofurantoin or norfloxacin in case of UTIs apart from cystitis. Nevertheless, the Baclet et al. criteria also referred to general principles of antibiotic use and the misuse for the treatment of specific organisms like for virus infections (11).

Demographic characteristics—i.e., age, sex, and International Statistical Classification of Diseases and Related Health Problems 10th Revision (ICD-10) diagnoses—were retrieved from patient records.

### 2.4 Statistical analysis

Continuous variables are depicted as means ± standard deviations (SDs). For categorical variables, absolute and relative frequencies were calculated. All statistical analyses were performed with IBM® SPSS® (Statistical Package for the Social Sciences) Statistics for Windows, version 29 (Armonk, New York, USA). Due to the retrospective character of our study, we opted for a conservative statistical approach and limited the statistical analyses to descriptive statistics.

## 3 Results

### 3.1 Study population and antibiotic prescriptions

**Figure 1:**
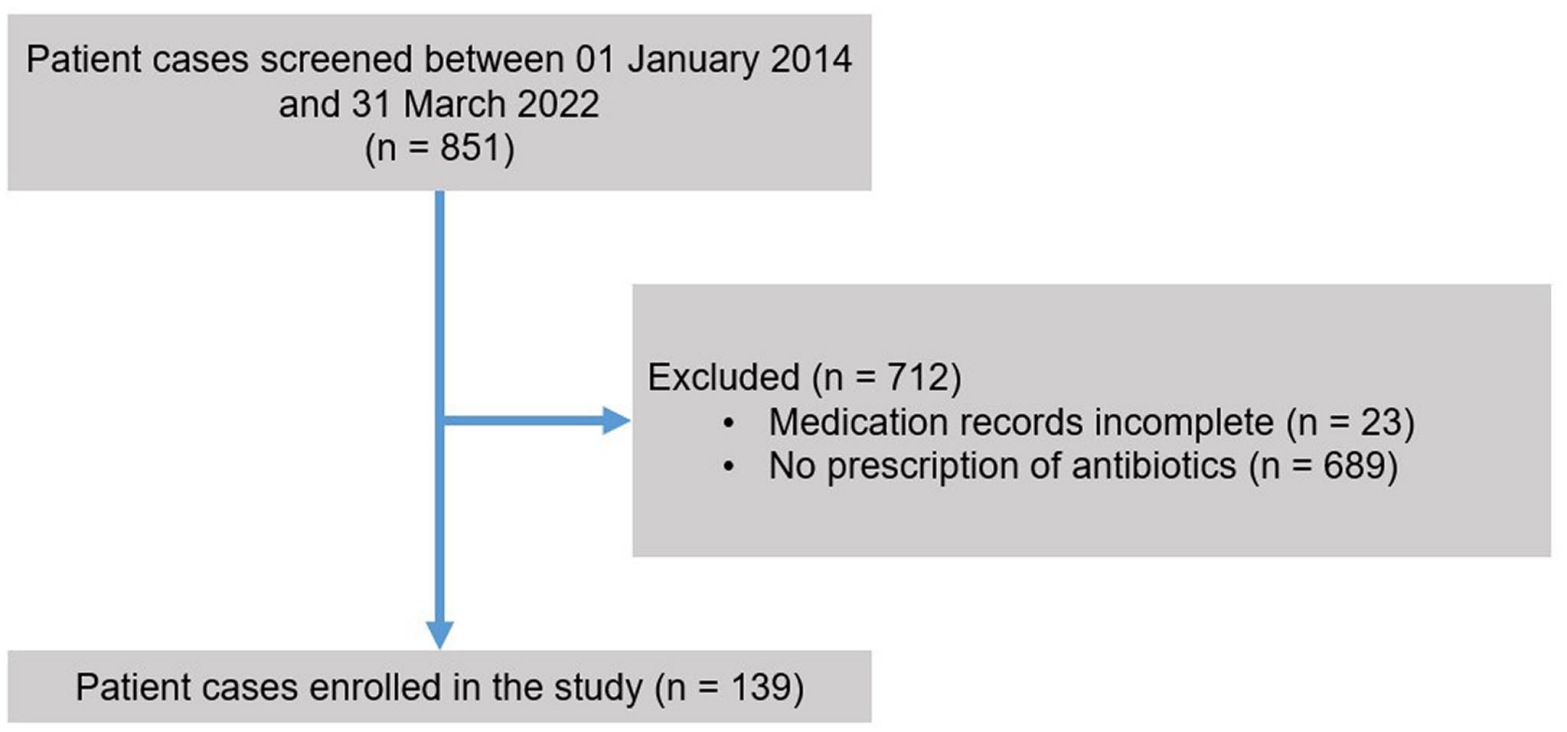
Flow of participants. Patients were eligible for inclusion in the study if they were treated on the geriatric psychiatric ward between January 2014 and March 2022 and received a course of antibiotic treatment (topical antibiotics were excluded). Patients needed to be at least 65 years of age and they or their legal guardian had to provide written informed consent.

139 patient cases involving 132 individual patients of 851 screened patient cases (685 individual patients) fulfilled the eligibility criteria and were enrolled in the study (Figure 1). The higher number of patient cases as compared to the number of individual patients is explained by returners. The mean age of the study population was 78.8 ± 8.2 years and 69.8% (97/139) of the patients were female (Table 1). Dementia was the most frequent psychiatric diagnosis in the study population (45.3%; 63/139), followed by delirium (34.5%; 48/139) and depression (31.7%; 44/139) (Table 1). The most prevalent somatic comorbidity was arterial hypertension, which affected 64.7% (90/139) of the study population (Table 1).

**Table 1:** Characteristics of the study population (*n* = 139).

Variables	*n*	%
Sex		
Female	97	69.8
Male	42	30.2
Psychiatric diagnoses^a^		
Depression^b^	44	31.7
Bipolar affective disorder^c^	8	5.8
Schizophrenia or schizophreniform disorder^d^	14	10.1
Mental and behavioral disorder due to use of alcohol, tobacco, or sedatives or hypnotics^e^	16	11.5
Dementia^f^	63	45.3
Delirium^g^	48	34.5
Other psychiatric disorder(s)	16	11.5
Somatic diagnoses^a^		
Arterial hypertension	90	64.7
Coronary heart disease	18	12.9
Chronic heart failure	33	23.7
Atrial fibrillation	29	20.9
Status post stroke	21	15.1
Type-2 diabetes mellitus	28	20.1
Chronic obstructive pulmonary disease	13	9.3
Hypothyroidism	20	14.4
Other somatic disorder(s)	127	91.4

The mean age ± standard deviation of the study population was 78.8 ± 8.2 years. ^a^Patients could have more than one diagnosis; ^b^ICD-10 F32, F33; ^c^ICD-10 F31; ^d^ICD-10 F06.2, F2X; ^e^ICD-10 F10, F13, F17; ^f^ICD-10 F00, F01, F02, F03; ^g^ICD-10 F05. ICD-10 denotes International Statistical Classification of Diseases and Related Health Problems 10th Revision.

In total, 195 antibiotic drugs were prescribed in the study population in 139 different patient cases, affecting 132 individual patients. Hence, the included patients with infectious diseases received 1.4 ± 0.8 courses of antibiotic treatment on average. An antibiotic treatment was prescribed in 16.3% (139/851) of all screened patient cases, involving 19.3% (132/685) of all treated patients in the study period. Penicillins were the most commonly prescribed antibiotics (35.9%; 70/195), followed by cephalosporins (24.1%; 47/195) and fosfomycin (16.9%; 33/195) (Table 2). 59.5% of antibiotic prescriptions were due to UTIs (116/195), followed by pulmonary infections (13.3%; 26/195) and skin and soft tissue infections (11.3%; 22/195) (Table 3). In the cases of UTIs, penicillins (27.6%; 32/116) fosfomycin (26.7%; 31/116) and cephalosporins (24.1%; 28/116) were most frequently used.

**Table 2:** Absolute and relative frequencies of antibiotic classes that were prescribed in the study population.

Antibiotic class	*n*	%
All prescribed antibiotics	195	100
Penicillins	70	35.9
Cephalosporins	47	24.1
Fosfomycin	33	16.9
Fluoroquinolones	21	10.8
Lincosamides	6	3.1
Nitroimidazoles	4	2.1
Carbapenems	4	2.1
Folic acid antagonists	3	1.5
Macrolides	2	1
Nitrofurantoin	2	1
Tetracyclines	1	0.5
Glycopeptides	1	0.5
Gyrase inhibitors	1	0.5

**Table 3:** Absolute and relative frequencies of infection sites that led to antibiotic treatment.

Antibiotic class	*n*	%
All considered sites of infection	195	100
Urinary tract	116	59.5
Pulmonary	26	13.3
Skin and soft tissue	22	11.3
Unclear focus	19	9.7
Gastrointestinal tract	9	4.6
Others	3	1.5

### 3.2 PIPs of antibiotics for older people according to the definition by Baclet et al.

53.3% (104/195) of all prescribed courses of antibiotic treatment were not identified as potentially inappropriate according to the classification by Baclet et al. (11). Apart from that, nearly half of all antibiotic treatments fulfilled at least one PIP criterium. 23.6% (46/195) of all antibiotic prescriptions involved one PIP criterion, 13.9% (27/195) involved two PIP criteria, and 9.2% (18/195) involved three or more PIP criteria. Overall, 177 PIPs could be validated by the expert panel. The most common PIP criterion was duration of an antibiotic course for more than seven days (15.3%; 27/177), followed by prescription of third-generation cephalosporins in cases of cystitis (11.9%; 21/177) and prescription of fluoroquinolones when third-generation cephalosporins could be used instead (10.8%; 19/177) (Table 4).

**Table 4:** Absolute and relative frequencies of potentially inappropriate prescription (PIP) categories of newly prescribed antibiotic drugs in our study population according to the classification by Baclet et al. (11).

PIP category	*n*	%
“It is potentially inappropriate to…”	177	100
Prescribe a course of antibiotics of more than 7 days	27	15.3
Prescribe a third-generation cephalosporin in case of cystitis	21	11.9
Prescribe a fluoroquinolone if a third-generation cephalosporin can be used	19	10.7
Prescribe ceftriaxone rather than cefotaxime when venous access is available	15	8.5
Prescribe fluoroquinolones as empirical therapy	14	7.9
Prescribe fluoroquinolones for the empirical therapy of urinary tract infections	13	7.3
Prescribe oral third-generation cephalosporins (except for documented switch in case of acute pyelonephritis in women)	13	7.3
Prescribe fluoroquinolones as a first-line treatment (apart from male urinary tract infections or acute pyelonephritis)	13	7.3
Prescribe fluoroquinolones for the first-line treatment of cystitis	11	6.2
Prescribe antibiotics for an isolated elevation of CRP	8	4.5
Prescribe antibiotics for urinary tract colonization	6	3.4
Prescribe a two-antibiotic combination in case of pneumonia	4	2.3
Prescribe amoxicillin-clavulanic acid for the empirical therapy of urinary tract infections	2	1.1
Prescribe antibiotics for the empirical therapy of diarrhea	2	1.1
Prescribe cotrimoxazole as empirical therapy (except when pneumocystosis is suspected)	2	1.1
Prescribe amoxicillin-clavulanic acid for male urinary tract infections	1	0.6
Prescribe a 3GC for Urinary tract infections	1	0.6
Prescribe any molecule other than amoxicillin for cellulitis of the lower limb	1	0.6
Fail to re-evaluate dosage according to renal function changes	1	0.6
Prescribe vancomycin without a loading dose	1	0.6
Prescribe a course of antibiotics of more than 7 days for pneumonia	1	0.6
Prescribe metronidazole for *Clostridium difficile* infections	1	0.6

## 4 Discussion

This study evaluated antibiotic use and screened for antibiotic PIPs in a cohort of geriatric psychiatric patients over a 99-months period. 16.3% of all screened patient cases received at least one antibiotic administration during hospitalization. Some patients received multiple antibiotic prescriptions during one hospital stay, with an average of 1.4 antibiotic courses per patient.

Patients affected by antibiotic prescriptions exhibited various somatic comorbidities, particularly cardiovascular diseases. As dementia and delirium were the leading psychiatric diagnoses (according to ICD-10) and also predispose for the development of ADRs, our study population can be classified as a high-risk group (13).

The use of antibiotics in older patients, in addition to the risk of development of multidrug-resistant strains, is associated with an increased risk of ADR occurrence. This most frequently concerns gastrointestinal ADRs, ranging from nausea and emesis to dangerous *Clostridium difficile* infections (14). Of note, probiotics were not routinely used as a preventative approach in our patient cohort. Since recent studies have shown a benefit in older patients, preventative use of probiotics should be reconsidered at our institution in the future (15). In addition to gastrointestinal symptoms, renal and cardiac antibiotic-related side effects are also common in older patients. In view of the high number of comorbidities and the vulnerability of our patient clientele, potential ADRs must be paid even more attention to. Likewise, neurologic and psychiatric side effects have also been described with antibiotic administration, although these are clearly less frequent and are estimated to have an incidence <1%. In our patient population, psychiatric ADRs have not been reported under antibiotic administration in our patient population, although a conclusive association is often difficult to confirm due to co-existing psychiatric and somatic disorders; thus, ADRs could have been masked (16).

With regard to the prevalence of antibiotic prescriptions in geriatric psychiatry, there have been few comparable studies to date (17, 18). Barman et al. examined the use of antibiotics on an acute geriatric psychiatric ward, with special emphasis on UTIs, and recorded a prescription rate of 27% (18). Ardoino et al., on the other hand, studied the use of antibiotics in geriatric patients in multiple hospitals, with a prevalence of antibiotic prescriptions during hospital stays of 48.2% (17). In contrast, the overall prevalence of antibiotic prescriptions was only 16.3% in our study population. The comparatively low prevalence of antibiotic prescriptions may be due to the fact that infections in the geriatric psychiatric setting are more likely to be comorbidities arising during inpatient treatment than leading admission diagnoses, and that severe infections are usually transferred to specialized internal medicine units. Furthermore, factors such as differing lengths of hospital stays as well as differences in inclusion criteria result in limited comparability of the data.

Regarding the infections that led to the use of antibiotics, there was a large proportion of UTIs (59.5%), followed by pulmonary (13.3%) and skin and soft tissue infections (11.3%). These findings are similar to the results reported by Barman et al., where UTIs were also the leading cause for antibiotic prescriptions with 53% (18). In contrast, the results of Ardoino et al. from internal medicine and geriatric hospitals showed pulmonary infections as the most common indication for antibiotic use, with UTIs in second place (17). In a study examining antibiotic prescriptions in patients with dementia, urinary tract and pulmonary infections were also found to be the most common infections leading to antibiotic use (19).

Due to the frequent use of antibiotics in geriatric psychiatry and the vulnerability of the patient population, it is important to screen for antibiotic PIPs. In our study, we found that 46.7% of all antibiotic prescriptions met at least one PIP criterium according to the definitions published by Baclet et al. (11). The most common PIP criterion was related to the duration of antibiotic prescription (prescription of an antibiotic longer than seven days).

Regarding the duration of antibiotic therapy, evidence clearly indicates that for most infectious diseases, shorter antibiotic treatment is as effective as longer antibiotic therapy (20, 21). Similarly, shortening antibiotic therapy results in fewer ADRs and reduces development of antibiotic resistance (2, 22). Although the administration of antibiotics for more than seven days may be necessary in well-justified cases, it should always be subject to careful consideration.

Other frequently identified PIP criteria were related to the use of antibiotics for UTIs. Here, PIPs concerned the administration of third-generation cephalosporins in cases of cystitis, the prescription of antibiotics in cases of asymptomatic bacteriuria, and the use of fluoroquinolones for UTIs. Regarding antibiotic treatment of UTIs, a general distinction must be made between complicated and uncomplicated UTIs, although classification systems are not consistent and are continuously evolving (23). Antibiotics such as fosfomycin are recommended in international guidelines for the treatment of uncomplicated cystitis in women (24). By contrast, cefpodoxime—also internationally recommended for uncomplicated UTI—is considered as potentially inappropriate in the Baclet et al. list (11). Encephalopathy, myoclonia, seizures, and delirium have been reported as ADRs for the use of cephalosporins, most commonly for cefepime, ceftazidime, cefuroxime, and cefazolin (25, 26). In particular, older patients with preexisting renal insufficiency and neurologic disorders are at risk (26). This especially applies to our geriatric psychiatric patient population, so that in the context of an uncomplicated UTI, the prescription of fosfomycin instead of cephalosporins appears preferable.

With regard to prescription of antibiotics in complicated UTIs, international guidelines predominantly recommend third-generation cephalosporins such as ceftriaxone or cefotaxime, as well as the use of fluoroquinolones (24). According to Baclet et al., the recommendations from international guidelines here predominantly represent PIPs (11). This demonstrates the difficulties for the selection of an appropriate antibiotic in geriatric psychiatric patients. Therefore, international guidelines should ideally incorporate separate recommendations for distinct patient populations.

Furthermore, five of the most frequently identified antibiotic PIPs in our study related to the prescription of fluoroquinolones. Reports of predominantly psychiatric and neurological ADRs of fluoroquinolones like mania, insomnia, acute psychosis, delirium or seizures have led to a more restrictive use in recent years (27). Especially the use of ciprofloxacin appears to be associated with an increased incidence of neurotoxic ADRs (26). In addition, it should be noted that fluoroquinolones increase the risk of cardiac arrhythmias by prolongation of the QT interval, which is important to consider especially due to frequent psychotropic polypharmacy in geriatric psychiatry, which may lead to the addition of QT interval-prolonging effects in the sense of a pharmacodynamic interaction. Therefore, the indication for the use of fluoroquinolones in geriatric psychiatric settings should be made with special caution (28).

Besides pharmacodynamic interactions between antibiotics and psychotropic drugs, pharmacokinetic interactions must also be taken into account. A notorious example in this regard is linezolid, which besides its antibiotic properties displays monoamine oxidase-inhibiting effects. Linezolid may thus interact with a plethora of psychotropic drugs, such as selective serotonin reuptake inhibitors, selective serotonin–norepinephrine reuptake inhibitors, tricyclic antidepressants, mirtazapine, trazodone, and tianeptine, only to name a few (29). If linezolid is combined with one of those drugs, a potentially lethal serotonin syndrome may arise. Another clinically relevant example is the interaction of macrolide antibiotics (e.g., clarithromycin), potent inhibitors of the cytochrome P450 isoenzyme (CYP) 3A4, and psychotropic drugs that are substrates of CYP3A4, e.g., quetiapine. Coadministration of macrolides and CYP3A4 substrates should be avoided (or is even contraindicated in some cases) due to the increased risk of side effects. Drug–drug interactions between antiinfectives (with a special emphasis on antibiotics) and second-generation antipsychotics have been comprehensively reviewed by Spina and colleagues (30).

Overuse of antibiotics was also a frequent topic in our study and has been detected in 14 cases: on the one hand, in cases of isolated CRP elevation, and on the other hand, when prescribing antibiotics in cases of asymptomatic bacteriuria. We already described the difficulties in the diagnosis of UTIs in older patients. In this context, the incorrect use of antibiotics in asymptomatic bacteriuria in older patients has been widely discussed in the literature (31). Overprescription leads to an increase in antibiotic resistance in UTIs in older patients, especially higher rates of resistance to fluoroquinolones or ß-lactam antibiotics compared to younger patient populations (31, 32).

Barman et al. studied PIPs in UTIs of geriatric psychiatric patients and found rampant overdiagnosis and overprescription of antibiotics for the treatment of asymptomatic bacteriuria (18). The authors estimate that 80% of geriatric psychiatric patients with a suspected UTI are overdiagnosed, leading to unindicated prescription of antibiotics with potentially severe ADRs as well as to premature rejection of other causes in the context of delirium diagnostics (18). Several studies have described the frequent misdiagnosis of UTIs in older patients, specifically identifying the overuse of urine tests as well as the misinterpretation of their results as a prominent cause. Similarly, errors in the collection of urine specimens in older patients are described (33, 34).

While fluoroquinolones were most commonly prescribed in cases of UTIs in the Barman et al. study, penicillins were used most frequently in our geriatric psychiatric patient population, followed by fosfomycin and cephalosporins (18). Reasons for this may be different local resistance situations and diverging awareness levels for the restricted use of fluoroquinolones, whereby different time periods of data collection should also be considered.

Although guidelines for the treatment of UTIs differ depending on regional contexts, the avoidance of fluoroquinolones is mentioned as one of the most frequent recommendations in international guidelines (35).

Regarding the PIP criterion “two-antibiotic combination in case of pneumonia,” four cases were recorded in our study. When it comes to the treatment of pneumonia, there are also different international guidelines, depending on whether pneumonias are considered as community-acquired or nosocomial. Concerning the use of combined antibiotic therapies, a meta-analysis by Kumar et al. found that antibiotic combinations may improve survival and clinical response in high-risk, life-threatening infections, but may have harmful effects in lower-risk patients (36). The most likely reason behind this is the toxicity of the drug, although other factors should also be considered, such as the development of resistant organisms (36). It should be mentioned that the antibiotic PIP criteria developed by Baclet et al. do not refer to severe disease courses (11). Furthermore, the severity of disease has not been captured in our data. In view of our vulnerable patient clientele, it is even more important to consider potential ADRs of antibiotics and to integrate them into the decision-making process for an appropriate antibiotic therapy.

In summary, our retrospective data analysis showed a frequent use of antibiotic PIPs in a geriatric psychiatric patient population. UTIs constituted the main reason for antibiotic prescriptions, followed by pulmonary infections. When using the Baclet et al. criteria, it should be noted that they were originally developed for patients aged 75 years and older, whereas we also applied the criteria to patients aged 65 years and older (11). Including these patients was a conscious decision as they constitute a representative part of the geriatric psychiatric patient population, and the criteria appeared equally suitable for this patient group. Moreover, the Baclet et al. criteria recommend excluding not clearly specified “severe courses” of infections (11). In our study, “severe courses” were not generally excluded, but the conditions of our geriatric psychiatric ward explicitly exclude intensive care treatment. Additionally, redundancy in the definitions of antibiotic PIPs according to Baclet et al. should be mentioned here (e.g., the double classification as PIP due to “use of fluoroquinolones as empiric therapy” and “use of fluoroquinolones as empiric therapy in UTIs”) (11). This has to be taken into account when evaluating the number of PIPs per antibiotic prescription. Furthermore, the recommendations of Baclet et al. do not represent an expert consensus, rather they should be considered expert recommendations (11). However, PIM lists commonly used in Germany, such as the PRISCUS 2.0 list and the FORTA classification, only give a few general statements on the use of antibiotic classes, but offer no specific criteria on their use for infectious diseases, the duration of antibiotic administration, or antibiotic combination therapies. While the FORTA classification provides a general risk–benefit assessment on different antibiotic classes, the PRISCUS 2.0 list only mentions fluoroquinolones, for which a restrictive use (independent of age) is recommended (9, 37).

Additionally, in geriatric psychiatric patients with frequent use of psychotropic medications with QT prolonging potential, special caution should be exercised when using QT interval prolonging antibiotics like fluoroquinolones or macrolides (38).

Limitations of our data result from the retrospective study design and possible differences in documentation quality of the prescribing physicians over the long period of data collection. Likewise, various factors such as diverging levels of expertise of the treating physicians, underlying diagnoses in patients, and the length of inpatient stays could not be adequately analyzed with regard to their influence on antibiotic prescriptions.

Due to the high number of patients included, we were able to systematically investigate antibiotic prescriptions in the field of geriatric psychiatry with regard to antibiotic PIPs for the first time. The results underscore the importance of critically examining the use of antibiotics in geriatric psychiatric patients and checking each antibiotic prescription for correct indication. Furthermore, the relevance of UTIs in the geriatric psychiatric setting became particularly apparent, showing the need for structured and validated recommendations for this patient group.

It would be advisable to establish a PIM list, compiled by expert consensus, which takes the use of antibiotics into sufficient account. Due to aging societies across the globe and an increasing relevance of psychiatric diseases, a targeted antibiotic management with few ADRs is essential for the treatment of infectious diseases. The establishment of recommendations for geriatric psychiatric patients in the future is therefore paramount.

## Data availability statement

The original contributions presented in the study are included in the article/supplementary material, further inquiries can be directed to the corresponding author.

## Ethics statement

The studies involving human participants were reviewed and approved by Ethics Committee of Hannover Medical School. The patients/participants provided their written informed consent to participate in this study.

## Author contributions

TP: Conceptualization, Data curation, Formal analysis, Methodology, Validation, Writing – original draft. SS: Validation, Writing – review & editing. JH: Validation, Writing – review & editing. SB: Validation, Writing – review & editing. TK: Validation, Writing – review & editing. FW: Validation, Writing – review & editing. AG: Conceptualization, Methodology, Validation, Writing – original draft. MSW: Conceptualization, Data curation, Formal analysis, Methodology, Supervision, Validation, Writing – original draft.
